# Embryonic stem cell differentiation requires full length Chd1

**DOI:** 10.1038/srep08007

**Published:** 2015-01-26

**Authors:** Paolo Piatti, Chin Yan Lim, Roxana Nat, Andreas Villunger, Stephan Geley, Yan Ting Shue, Claudia Soratroi, Markus Moser, Alexandra Lusser

**Affiliations:** 1 Division of Molecular Biology, Biocenter, Medical University of Innsbruck, Austria; 2 Epithelial Epigenetics and Development Laboratory, Institute of Medical Biology, A*Star, Singapore; 3 Institute for Neuroscience, Medical University of Innsbruck, Austria; 4 Division of Developmental Immunology, Biocenter, Medical University of Innsbruck, Austria; 5 Division of Molecular Pathophysiology, Biocenter, Medical University of Innsbruck, Austria; 6 Department of Molecular Medicine, Max Planck Institute of Biochemistry, Martinsried, Germany

## Abstract

The modulation of chromatin dynamics by ATP-dependent chromatin remodeling factors has been recognized as an important mechanism to regulate the balancing of self-renewal and pluripotency in embryonic stem cells (ESCs). Here we have studied the effects of a partial deletion of the gene encoding the chromatin remodeling factor Chd1 that generates an N-terminally truncated version of Chd1 in mouse ESCs *in vitro* as well as *in vivo*. We found that a previously uncharacterized serine-rich region (SRR) at the N-terminus is not required for chromatin assembly activity of Chd1 but that it is subject to phosphorylation. Expression of Chd1 lacking this region in ESCs resulted in aberrant differentiation properties of these cells. The self-renewal capacity and ESC chromatin structure, however, were not affected. Notably, we found that newly established ESCs derived from *Chd1^Δ2/Δ2^* mutant mice exhibited similar differentiation defects as *in vitro* generated mutant ESCs, even though the N-terminal truncation of Chd1 was fully compatible with embryogenesis and post-natal life in the mouse. These results underscore the importance of Chd1 for the regulation of pluripotency in ESCs and provide evidence for a hitherto unrecognized critical role of the phosphorylated N-terminal SRR for full functionality of Chd1.

Eukaryotic chromatin is a highly organized structure that serves to compact the long linear DNA molecules in the cell nucleus. At the base of the structural hierarchy lies the nucleosome, which is the fundamental repeating unit of chromatin and is composed of 147 bp of DNA wrapped around an octamer of the basic histone proteins H2A, H2B, H3 and H4^1^. The organization of chromatin in the nucleus is not uniform but consists of areas with distinct features often reflecting the functional state of the region. The highly condensed heterochromatin comprises areas with low transcription activity while the more “open” euchromatin is highly permissible for transcription^2^. The relative proportions of these chromatin states can vary greatly in different cell types. Stem cells, such as ESCs, typically contain large euchromatic areas that are accessible for nuclear proteins (“hyperdynamic chromatin”). As cells differentiate, increasing heterochromatinization is observed^3^,^4^. ESCs are derived from the inner cell mass of the mammalian blastocyst. They have the capacity for indefinite self-renewal and are pluripotent. The unique chromatin conformation in ESCs is considered to be a major determinant of pluripotency^4^. The predominantly euchromatic nature of ESCs is reflected by relative enrichment of histone modification marks that are usually associated with transcriptional activity, such as histone acetylation or methylation of H3 lysine 4 (H3K4me3) and lysine 36 (H3K36me2/3)^3^,^5^. Moreover, this particular open chromatin configuration seems to be responsible for the maintenance of the ESC genome in a transcriptionally hyperactive state, in which even normally silenced repetitive elements as well as coding and non-coding regions are transcribed^3^. Nevertheless, differentiation-associated genes are repressed and exhibit a “bivalent” chromatin signature with simultaneous presence of the activity-related H3K4me3 and the repressive H3K27me3 marks, which are resolved upon differentiation to either the active or repressive mark according to lineage requirements^6^,^7^.

The importance of chromatin modifying factors for maintaining the balance between self-renewal and pluripotency of ESCs has become increasingly clear in recent years^4^,^8^,^9^,^10^,^11^. Energy-dependent alteration of histone-DNA contacts within eukaryotic chromatin is one of the major strategies to influence chromatin structure and function. This process is catalyzed by dedicated ATPases belonging to the SWI/SNF superfamily^12^,^13^ and can involve the loading of histones onto the DNA to form a nucleosome, the removal of part or all of the nucleosomal histone core, histone exchange, as well as repositioning of the histone core along the DNA sequence^14^,^15^,^16^. In concert with other chromatin modifying mechanisms, such as modifications of histones and DNA, the incorporation of histone variants or the action of non-coding RNAs, ATP-dependent chromatin remodeling is able to dynamically shape local and global chromatin structure and compaction and thereby contributes to the regulation of all processes that require access to the DNA sequence. Several ATP-dependent chromatin remodeling factors have critical functions in ESCs. For example, the ESC-specific esBAF complex, which contains the Brg1 ATPase along with the ESC-specific BAF155 and BAF60A subunits, localizes to a considerable number of genes encoding master regulators, such as Oct4 and Sox2, and is required for self-renewal^17^,^18^. The Tip60-p400 complex, which contains a histone acetyltransferase in addition to the motor subunit p400, was shown to be necessary for self-renewal and pluripotency in ESCs and functions in concert with Nanog to regulate ESC-specific and developmental genes^19^. NuRD is another chromatin remodeling complex that contains both histone modifying (histone deacetylase) and ATP-dependent remodeling enzymes (Chd3 or Chd4). ESCs lacking the Mbd3 subunit of NuRD failed to downregulate pluripotency markers during embryoid body (EB) formation and could not commit to specific developmental programs^20^. NuRD is also required for the proper transcriptional balance of self-renewal factors in ESCs^21^. Similarly, the SNF2L containing complex NURF appears to have developmental roles, as ESCs lacking the largest subunit Bptf exhibit severe defects in the formation of meso- and endoderm and to a lesser degree of ectodermal cell fates, and *Bptf* null embryos die between embryonic day E7.5 and E8.5^22^. Very recently, it was shown that the chromatin remodeler Ino80 regulates pluripotency gene expression and is required for ESC self-renewal^23^.

The ATP-utilizing SNF2 family member Chd1 is a chromatin remodeling and assembly factor^24^,^25^. Knock-down of *Chd1* in ESCs was shown to result in increased differentiation into the ectodermal lineage at the expense of mesoderm and endoderm but had only minor effects on self-renewal of the ESCs^26^. Chd1 has been linked to active transcription in a number of organisms due to its colocalization with RNA polymerase II or H3K4me3^26^,^27^,^28^,^29^ and its interaction with transcription initiation, elongation and splicing factors^30^,^31^,^32^,^33^,^34^. Interestingly, RNAi-driven *Chd1* knock-down in ESCs caused the appearance of more compact heterochromatin foci than in control ESCs, and it was proposed that a global increase in heterochromatin may be responsible for the differentiation defects in the knock-down cells^26^.

In this work we set out to investigate the effects of a complete deletion of *Chd1* on self-renewal and differentiation potential of mouse ESCs. To this end, we targeted the start codon-containing exon 2 of the *Chd1* gene for Cre recombinase-mediated deletion. This strategy resulted in the production of an N-terminally truncated mutant Chd1 protein, which lacks a particularly serine-rich region (SRR), instead of the predicted loss of Chd1. The N-terminal SRR is present in all higher eukaryotic Chd1 proteins but so far its role for Chd1 activity at the cellular and organismal level has not been studied. We report here on the biochemical and genetic characterization of the N-terminally truncated mutant Chd1 (Chd1ΔSRR). Our study demonstrates that the SRR is subject to phosphorylation, and it provides evidence that loss of the SRR renders Chd1 unable to support normal differentiation of ESCs into the three germ layers *in vitro*. We show that *in vivo*, developmental defects caused by the Chd1ΔSRR mutation can be compensated and are compatible with normal life and development of the animal.

## Results

### Targeting the mouse *Chd1* gene locus and generation of *Chd1^Δ2/Δ2^* ESCs

**Figure 1:**
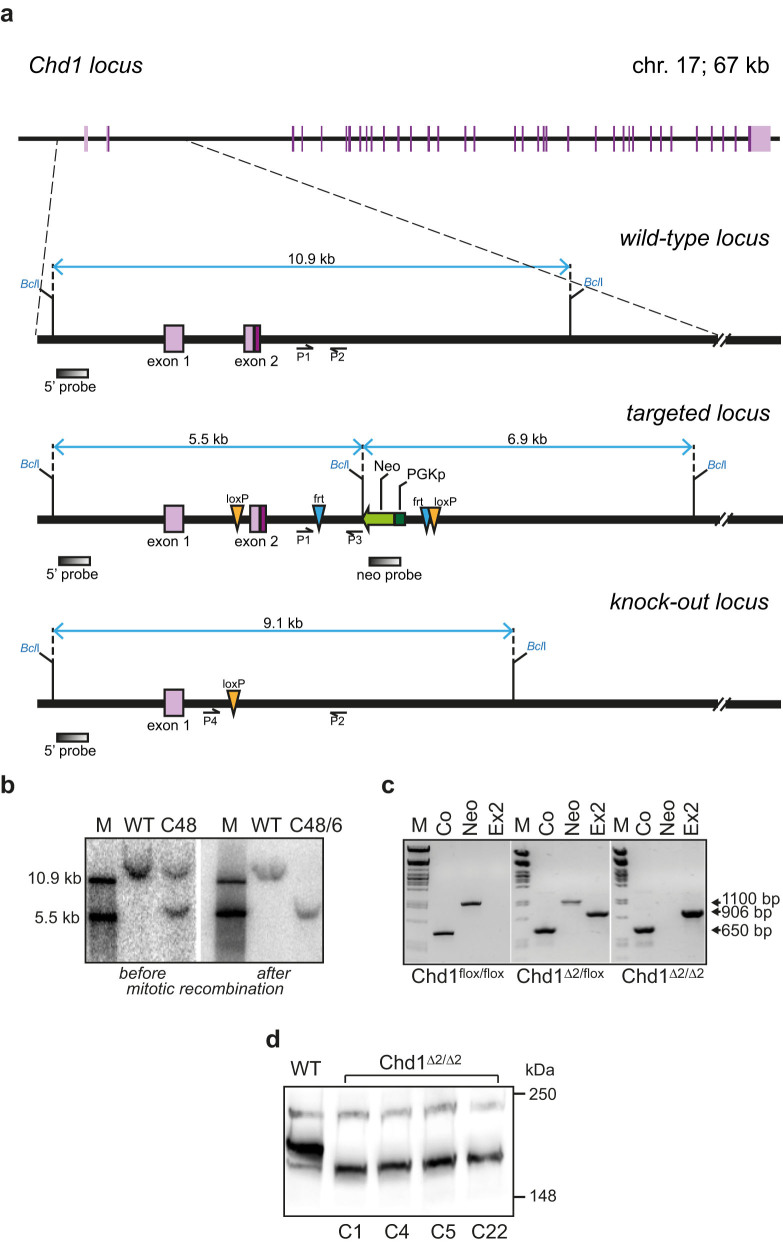
Generation and characterization of double floxed and *Chd1^Δ2/Δ2^* ESC lines. (a) Schematic representation of the wild-type, targeted and knock-out *Chd1* locus. The targeting construct contains exon 2 flanked by loxP recombination sites and a neomycin resistance cassette (Neo) controlled by the phosphoglycerate kinase 1 promoter (PGKp). P1-P4, locations of PCR screening primers; gray boxes, location of Southern blot probes. (b) Southern blot analysis of the *Chd1* locus in wild-type (wt) ESCs and in a targeted clone (C48) before mitotic recombination (left panel; the two bands indicate the presence of one wt and one targeted allele) and after successful mitotic recombination (only the band corresponding to the targeted allele is detected in C48/6). (c) PCR genotyping results confirm successful deletion of exon 2 after Cre-induction in clone C48/6. Co, control PCR product from outside the targeting region; Neo, neo-cassette specific product; Ex2, primers flanking the targeting cassette (P4+P2). (d) Western blot of protein extracts from wt ESCs and four independent *Chd1^Δ2/Δ2^* clones. Anti-Chd1 antibodies detect a faster migrating band in the *Chd1^Δ2/Δ2^* clones compared to the wt.

To study the effects of a complete deletion of Chd1 on self-renewal and pluripotency of ESCs, we targeted exon 2 of the *Chd1* gene in ESCs and used mitotic recombination to obtain homozygously targeted ESC clones. In the targeting construct exon 2 was flanked by loxP sites to allow conditional deletion by Cre-mediated recombination (Figure 1a). We obtained six clones exhibiting successful homologous recombination without additional ectopic integration of the targeting construct (Figure 1b). Clone C48 was further subjected to high concentration G418 selective pressure to induce mitotic recombination events, which produced a clone in which both *Chd1* alleles contained the floxed exon 2 (*Chd1^flox/flox^*; Figure 1b). Following the introduction of tamoxifen-inducible Cre recombinase^35^ and activation of Cre by tamoxifen treatment, deletion of exon 2 was verified by PCR and Southern blotting. Figure 1c shows a representative image of genotyping results for a *Chd1^flox/flox^*, a heterozygously and a homozygously deleted ESC clone (*Chd^flox/Δ2^, Chd1^Δ2/Δ2^*). Deletion of exon 2, which harbors the translational start codon, was expected to result in abrogated Chd1 protein production, since the subsequent ATG located in exon 3 was predicted to yield only a short out-of-frame polypeptide. However, when we analyzed protein extracts from *Chd1^Δ2/Δ2^* cells, we detected a shorter Chd1 antibody-reactive band indicating that an ATG located even further downstream was used for the production of an N-terminally truncated Chd1 (Figure 1d). Thus, exon 2 deletion gave rise to a hypomorphic allele.

### Biochemical characterization of the N-terminally truncated *Chd1* protein

**Figure 2:**
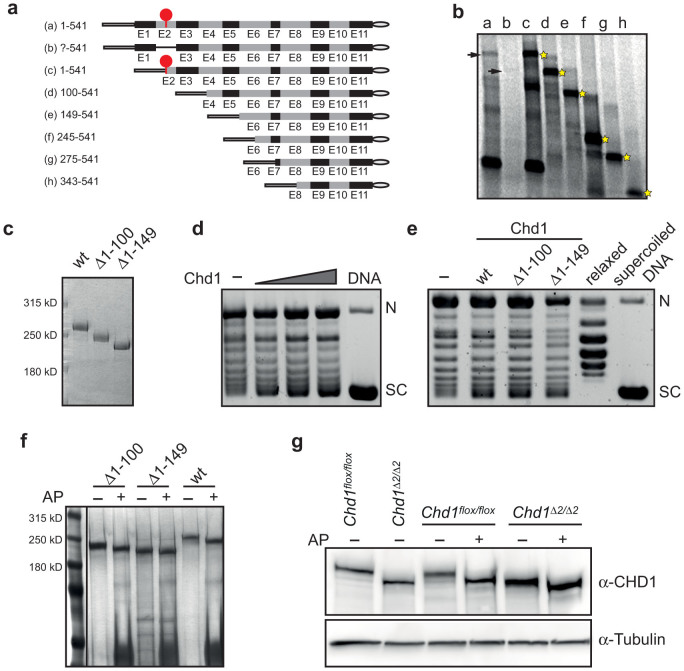
N-terminally truncated Chd1 is active in nucleosome assembly but lacks major phosphorylation sites. (a) DNA templates used for *in vitro* transcription/translation experiments. All sequences contain a Flag-tag at the end of exon 11. *a*, DNA fragment corresponding to the wt sequence including the natural transcription start site; *b*, DNA fragment corresponding to the transcript of the exon 2-deleted *Chd1* locus; *c-h*, DNA fragments starting with in-frame ATGs located in downstream exons of the *Chd1* gene. The red dot depicts the natural translation start codon. (b) Deletion of exon 2 results in protein translation starting at an ATG in exon 4. ^35^S-labeled *in vitro* translation products were separated by SDS-PAGE and visualized by phosphoimaging. Stars depict Chd1 polypeptides used as size markers; arrows indicate translation products of templates *a* and *b*. (c) WT and N-terminal truncation mutants of Chd1 were expressed using the baculovirus system, purified and separated by gel electrophoresis and Coomassie Blue staining. (d) Mouse Chd1 catalyzes the assembly of nucleosomes on relaxed circular DNA in a dose dependent manner. Chromatin assembly reactions were carried out with increasing amounts (5, 10, 20 nM) of recombinant wt Chd1, and DNA supercoiling was analyzed after 70 min. (e) N-terminally truncated Chd1 is capable of efficient nucleosome assembly. Products of nucleosome assembly reactions with wt, Δ1-100 and Δ1-149 proteins (20 nM) were analyzed for DNA supercoiling efficiency. Reference lanes contain topoisomerase I-relaxed (*relaxed*) and supercoiled plasmid DNA (*supercoiled*), respectively. The positions of nicked (N) and fully supercoiled (SC) DNA species are indicated. (f) Chd1 is phosphorylated at the N-terminal SRR. Recombinant wt, Δ1-100 and Δ1-149 proteins were treated with alkaline phosphatase (AP), separated by SDS-PAGE and stained with silver. (g) Mouse Chd1 is a phosphoprotein *in vivo*. Treatment of protein extracts from *Chd1^flox/flox^* cells with AP caused a mobility shift of Chd1 in SDS-PA gel electrophoresis, which was less pronounced for Chd1ΔSRR extracted from *Chd1^Δ2/Δ2^* cells. Chd1 was detected by immunoblotting with anti-Chd1 antibodies. Alpha-tubulin was used as a loading control.

To determine which ATG gives rise to the truncated protein, we generated a panel of PCR fragments containing a T7 promoter sequence fused to different potential translational start codons along the *Chd1* sequence (fragments *c-h* in Figure 2a). *In vitro* transcription followed by translation of these templates resulted in a protein ladder that we used for size comparison with the products obtained from *in vitro* translation of sequences containing the native 5′UTR encoded by exons 1 and 2 as well as the UTR that is generated by deletion of exon 2 (fragments *a* and *b* in Figure 2a). Figure 2b shows that protein production from the 5′-UTR containing templates was weak compared to the templates in which the start codon follows the T7 promoter sequence with only a short spacer. This is probably due to the increased length of the untranslated region. Nevertheless, the wild-type mRNA (fragment *a*) gave rise to a protein of equal size to that of template *c*, which contains the natural start codon. Although the sequence corresponding to the mutated mRNA (fragment *b*) turned out to be a very poor template for the *in vitro* reaction, a weak signal was detected at the size of the fragment *d* product (arrow in Figure 2b). This result together with the observed size difference of the native proteins on Western blots (Figure 1d) and size comparison with recombinant N-terminally truncated Chd1 proteins (Figure 2c) suggest that the first in-frame AUG downstream of the natural start codon is used in cells upon deletion of exon 2. The new start codon is located in exon 4 and results in the generation of a protein lacking the first 100 amino acids, thereby deleting most of a particularly serine-rich region (SRR) in the N-terminus, while retaining the functionally important chromodomains and the ATPase domain.

To determine if the loss of the SRR affects the biochemical activity of Chd1, we performed *in vitro* chromatin assembly assays of wild-type Chd1 as well as of two N-terminal deletion mutants that were generated using the baculovirus expression system. Mutant protein Δ1-100 corresponds to the truncated protein observed after *in vivo* deletion of exon 2 while Δ1-149 lacks the entire SRR (Figure 2c). DNA supercoiling analysis, which allows for the estimation of nucleosome formation efficiency in relaxed circular DNA, revealed concentration-dependent nucleosome assembly activity of recombinant murine Chd1 (Figure 2d). When we tested the mutant proteins in this assay, no significant decrease in the extent of DNA supercoiling was observed (Figure 2e). Furthermore, nucleosome assembly activity of wild-type as well as mutant proteins was dependent on the presence of ATP (Supplementary Figure S1). These results indicate that the SRR at the N-terminus is not required for nucleosome assembly by Chd1.

### The SRR of Chd1 is phosphorylated *in vivo*

Given the high number (35%) of serine residues within the first 100 amino acids, we reasoned that this region might be subject to phosphorylation, which in turn could regulate the functional properties of the protein. *In silico* analysis using the NetPhos2.0 algorithm predicted at least 29 potential serine phosphorylation sites within the first 100 amino acids and 41 within the first 150 amino acids. We therefore treated the recombinant wild-type and N-terminally truncated proteins with alkaline phosphatase (AP) and monitored potential mobility shifts by SDS-PAGE. Indeed, AP treatment resulted in faster migrating protein species for the wild-type and to a lesser extent the Δ1-100 protein, while dephosphorylation had only minor effects on the Δ1-149 protein (Figure 2f). AP treatment of protein extracts derived from *Chd1^flox/flox^* and *Chd1^Δ2/Δ2^* cells and subsequent immunoblotting revealed similar mobility shifts of endogenous Chd1 (Figure 2g). These results allow for the following conclusions: (i) The SRR of Chd1 is phosphorylated *in vivo*; (ii) The first 100 amino acids harbor a portion of the phosphorylated sites, and (iii) there is additional phosphorylation in the 49 amino acids of the SRR that remain intact in the Chd1ΔSRR protein.

**Figure 3:**
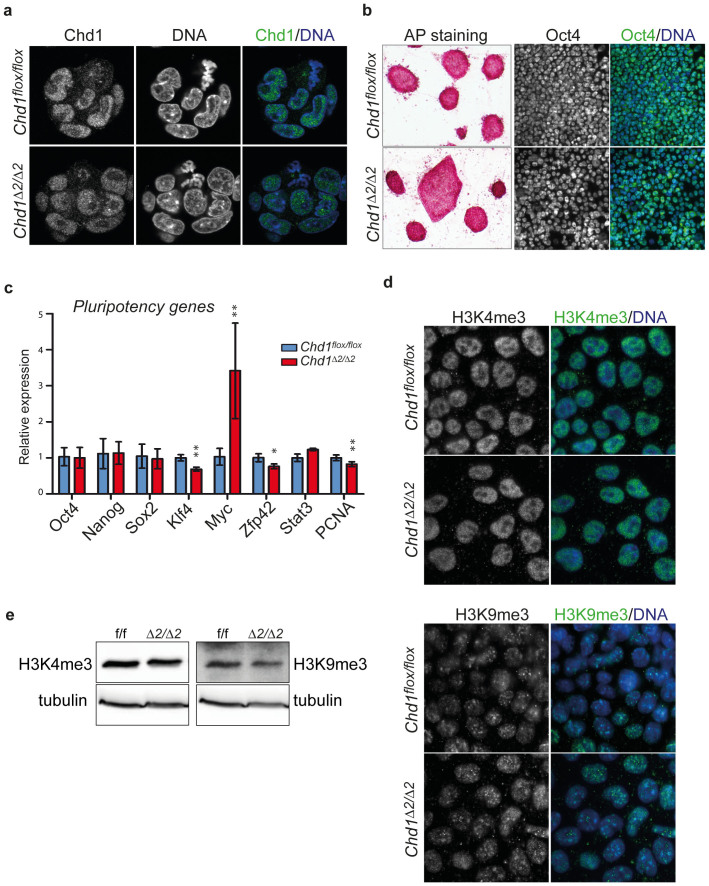
N-terminal truncation of Chd1 does not affect its intracellular localization, overall chromatin structure or regulation of pluripotency genes in ESCs. (a) Immunostaining of Chd1 in control (*Chd1^flox/flox^*) and *Chd1^Δ2/Δ2^* ES cells. (b) Alkaline phosphatase (AP) staining (left panels) and Oct4 (green) staining of wild-type and mutant ESCs. (c) The expression of pluripotency genes is largely unaffected in *Chd1^Δ2/Δ2^* ESCs. RT-qPCR was performed on cDNA derived from *Chd1^flox/flox^* and *Chd1^Δ2/Δ2^* ESC clones. Transcript levels of ESC marker genes were normalized against *Gapdh* and are expressed relative to those of the control line (*Chd1^flox/flox^*). Values represent mean +/- SD of 2-3 experiments each with two independent *Chd1^flox/flox^* and *Chd1^Δ2/Δ2^* ESC clones (*p<0.05; **p<0.001). (d) Distribution of euchromatin and heterochromatin appears normal in *Chd1^Δ2/Δ2^* cells. *Chd1^flox/flox^* control and *Chd1^Δ2/Δ2^* ESCs were stained with antibodies against H3K4me3 or H3K9me3. DNA was visualized by DAPI. (e) Bulk H3 methylation levels are comparable in *Chd1^flox/flox^* and *Chd1^Δ2/Δ2^* ESC clones. Whole cell protein extracts were subjected to SDS-PAGE and Western blotting and incubated with antibodies against H3K4me3 or H3K9me3 and tubulin (loading control).

Phosphorylation of the SRR of Chd1 might have different consequences. For instance, it might affect its intracellular localization behavior, interaction with other proteins or protein stability. To determine, if deletion of the SRR – thereby removing more than two thirds of the potential phosphorylation sites - affects the intracellular localization properties of Chd1, we performed immunostaining with Chd1 antibodies in floxed and mutant cells. Chd1ΔSSR showed the typical nuclear localization during interphase and loss of chromatin association during mitosis similar to the wild-type protein (Figure 3a). In addition, when we treated isolated nuclei with different salt concentrations, no differences in the elution of wild-type and Chd1ΔSSR were observed (Supplementary Figure S2). Thus, the SRR does not affect nuclear localization or overall chromatin affinity of Chd1.

### N-terminal truncation of Chd1 does not interfere with expression of ESC marker genes

To examine if removal of the phosphorylated SRR of Chd1 affects its activity in ESCs, we first examined cell proliferation rate and cell cycle distribution by cell counting and FACS analysis. These analyses revealed no differences between mutant and floxed ESCs (data not shown). In addition, alkaline phosphatase activity was not visibly different in *Chd1^Δ2/Δ2^* and *Chd1^flox/flox^* ESCs (Figure 3b). Previously, it was reported that RNAi-mediated knock-down of *Chd1* resulted in compromised pluripotency of ESCs^26^. To determine, if this is also the case in the presence of an N-terminally truncated Chd1, we performed reverse transcription quantitative PCR (RT-qPCR) for a panel of ESC markers. These experiments revealed no or minor (e.g. *Klf4, Zfp42*) changes in the transcript levels of most of the major pluripotency genes tested (Figure 3c), with the exception of *Myc* that was about 3fold upregulated in *Chd1^Δ2/Δ2^* cells. Similar expression of *Oct4* in mutant and wild-type ESCs was also evident in immunostainings (Figure 3b). To detect potential changes in overall chromatin appearance and distribution of euchromatin and heterochromatin, we performed immunostaining with antibodies against methylated histones. We did not observe obvious differences in the pattern or intensity of H3K4me3 (euchromatin) or H3K9me3 (heterochromatin) staining between *Chd1^Δ2/Δ2^* and *Chd1^flox/flox^* ESCs (Figure 3d). Likewise, immunoblotting of bulk H3K9me3 and H3K4me3 revealed comparable levels of modification in both cell lines (Figure 3e). Thus, it appears that loss of the N-terminal domain does not incapacitate Chd1 to an extent that results in the increased heterochromatin formation shown before in *Chd1* knock-down ESCs^26^. Together these results suggest that the SRR at the N-terminus of Chd1 is not required for ESC survival and self-renewal, that it does not affect overall chromatin appearance and that pluripotency gene expression is largely undisturbed.

### The N-terminal region of Chd1 is important for ESC differentiation

**Figure 4:**
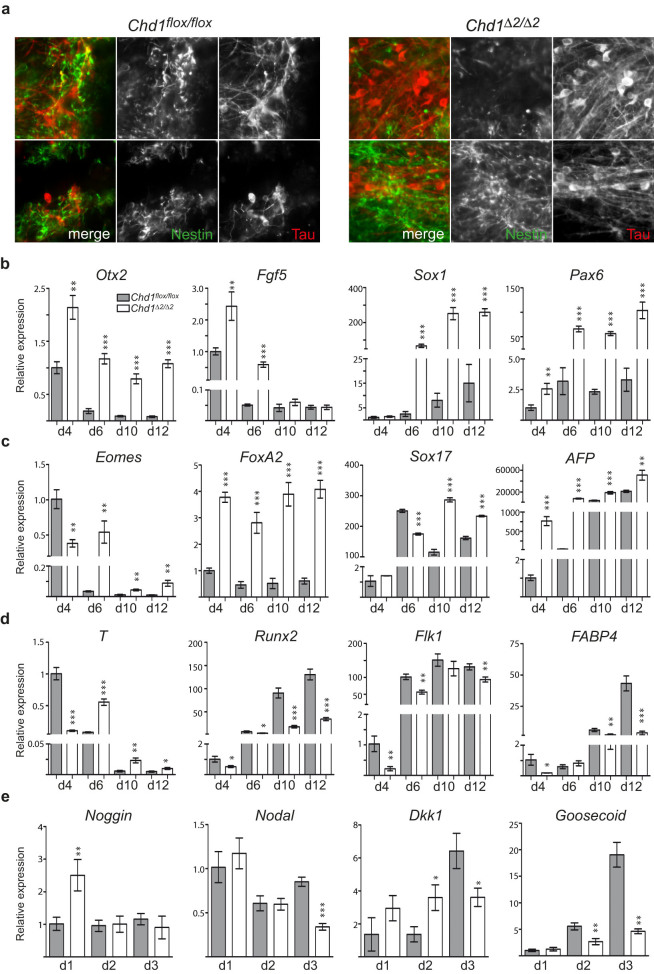
Mutant Chd1 protein causes altered ESC differentiation and expression of germ-layer marker genes. (a) *Chd1^Δ2/Δ2^* EB outgrowths show enhanced appearance of neural structures. *Chd1^flox/flox^* and *Chd1^Δ2/Δ2^* ESCs were induced to form EBs using the hanging drop method. After two days EBs were seeded on gelatin-coated 6-well plates for further culturing and stained with antibodies against the neural progenitor marker Nestin (green) and the neuronal marker Tau (red) at day 14. (b-d) *Chd1^Δ2/Δ2^* EB outgrowths show increased expression of ectodermal and endodermal marker genes and reduced levels of mesoderm markers. RT-qPCR was performed on cDNA derived from outgrowing *Chd1^flox/flox^* and *Chd1^Δ2/Δ2^* EBs at the indicated time points and the expression levels of the indicated ectoderm (b), endoderm (c) and mesoderm (d) marker genes were determined. (e) Signaling pathway components are misregulated in *Chd1^Δ2/Δ2^* early differentiating ESCs. RT-qPCR analyses were performed as in (b-d) for the indicated signaling pathway components. Transcript levels were normalized against *Gapdh* and are expressed relative to those of control EB outgrowths at day 4 (*Chd1^flox/flox^*). Values represent mean +/- SD of 2-3 experiments each with outgrowing EBs derived from two independent *Chd1^flox/flox^* and *Chd1^Δ2/Δ2^* ESC clones (*p<0.05; **p<0.001; ***p<0.0001).

Although unaltered regulation of stem cell markers in ESCs might point towards intact pluripotency of *Chd1^Δ2/Δ2^* cells, potential defects may become obvious only upon differentiation. Therefore, we induced two independently derived *Chd1^Δ2/Δ2^* and *Chd1^flox/flox^* ESC lines to differentiate into EBs using the hanging drop method for 2 days and further monitored outgrowth of differentiating EBs plated on gelatin-coated plates over a time period of 12 days. Intriguingly, *Chd1^Δ2/Δ2^* EB-derived outgrowths exhibited remarkable differences in their morphology from *Chd1^flox/flox^* EBs. In particular, we observed a higher prevalence of neuron-like structures in *Chd1^Δ2/Δ2^* outgrowths, which became visible at early time points of differentiation, when scarcely any such structures were observed in the *Chd1^flox/flox^* controls, and they developed into elaborate networks at later time points of EB outgrowth. By contrast, smaller and more localized neural structures were formed in *Chd1^flox/flox^* controls. Example images of stainings with the neural progenitor marker Nestin and the neuronal marker Tau on day 14 outgrowths are shown in Figure 4a and Supplementary Figure S3. Thus, the SRR is important for the functions of Chd1 in ESC differentiation.

### Disturbed germ-layer formation in differentiating *Chd1^Δ2/Δ2^* ESCs

To study the molecular features underlying perturbed ESC differentiation upon *Chd1* mutation, we analyzed the expression levels of various marker genes of the three germ layers at different time points of differentiation. As shown in Figure 4b, ~100 to 200 fold upregulation of early neurectoderm markers *Pax6* and *Sox1* was observed starting from day 6 in the differentiation of *Chd1^Δ2/Δ2^* ESCs. Transcript levels of *Pax6* and *Sox1* were significantly higher than levels in control cells, indicating strong overrepresentation of neuroectodermal cells in mutant EB colonies. Also the neural progenitor marker *Nestin* as well as *Doublecortin*, which is expressed in neuronal precursor cells and immature neurons, showed higher expression in the mutant EBs (Supplementary Figure S4). These expression patterns complement the staining results from Figure 4a and confirm the robust neural differentiation observed in *Chd1^Δ2/Δ2^* EB outgrowths. The neuroectodermal transcription factor *Otx2*, which is an early responding factor of ESC differentiation and was shown to precede formation of Sox1^+^ cells^36^ was clearly upregulated in day 4 *Chd1^Δ2/Δ2^* EB outgrowths and remained high throughout differentiation (Figure 4b). We found a slight but significant downregulation of *Nanog* in day 4 *Chd1^Δ2/Δ2^* EB colonies compared to *Chd1^flox/flox^* colonies (Supplementary Figure S4) and higher initial levels of the epiblast marker *Fgf5* (Figure 4b). These results are consistent with earlier studies reporting that increased *Otx2* expression leads to increased *Fgf5* expression and repression of *Nanog*^36^.

Analysis of the primitive streak (PS) markers *Brachyury* (*T*), *Eomes* and *FoxA2*^37^ revealed misregulation of these factors. The expression patterns of both *T* and *Eomes* suggested delayed upregulation in *Chd1^Δ2/Δ2^* cells compared to control colonies (Figure 4c, d), which was confirmed by analysis of earlier (days 1-3) EB differentiation time points (Supplementary Figure S5). In the embryo, PS expression of *Eomes* and *T* leads to formation of definitive endoderm and cardiac mesoderm structures^37^,^38^. Indeed, mesoderm-specific marker genes, such as *Runx2*, *Fabp4* and *Flk1*, showed reduced expression in mutant differentiating ESCs (Figure 4d) suggesting weaker and/or delayed mesoderm formation.

We also observed that the endoderm marker *FoxA2* was strongly upregulated in differentiating *Chd1^Δ2/Δ2^* ESCs at all time points tested. Consistent with this, we found greater upregulation of the endoderm marker *Sox17* in mutant cells at later time points (Figure 4c). However, downregulation of *Goosecoid* (*Gsc*), which is expressed in definitive endoderm, and high levels of *Afp* suggested the enhanced presence of cells with a visceral endoderm signature (*Sox17^+^Gsc^−^*)^39^ instead of definitive endoderm (*Sox17^+^Gsc^+^*; Figure 4c, e). Transcript levels of the master regulators of endoderm formation, *Gata4* and *Gata6*, however, were slightly but consistently lower in *Chd1^Δ2/Δ2^* EB colonies compared to control EB colonies at the time points tested (Supplementary Figure S4). This may point towards perturbed primitive endoderm formation or, since both factors are also expressed at later developmental stages in cardiac cell progenitors and their expression is linked to cardiomyocyte specification^40^,^41^, it may reflect decreased mesoderm formation as suggested also by the lower or delayed expression levels of other mesoderm markers (Figure 4d). Interestingly, we also found that components of the BMP, TGFβ and Wnt signaling pathways (*Nodal, Gsc, Noggin, Dkk1*) displayed altered regulation in differentiating *Chd1^Δ2/Δ2^* ESCs compared to control ESCs during the first three days of differentiation (Figure 4e). Of note, expression of *Noggin* was significantly higher in undifferentiated *Chd1^Δ2/Δ2^* ESCs compared to control ESCs (Supplementary Figure S5). Taken together, these data suggest that full-length Chd1 may be involved at the earliest time points of ESC differentiation by regulating the formation of primitive endoderm and epiblast and that it may further play a critical role in the specification of mesendoderm cell fates.

### Effects of Chd1 truncation on early development are compensated in the animal

The observed effects of the absence of the SRR of Chd1 on ESC differentiation prompted us to ask if embryonic development is affected in a similar way *in vivo*. To this end, we injected several ES cell clones bearing monoallelic floxed exon 2 into mouse blastocysts. Of two obtained chimeric mice, one displayed germline contribution and allowed for the establishment of a homozygous *Chd1^flox/flox^* line. These mice were bred to *Ubi-Cre* mice to remove exon 2. Contrary to expectation, normal mendelian distribution was observed in the progeny of *Chd1^Δ2/+^* heterozygous intercrosses (Supplementary Figure S6). The *Chd1^Δ2/Δ2^* mice, which expressed the truncated Chd1ΔSSR protein in all tissues tested (Supplementary Figure S6), appear healthy and are comparable in size and weight to their wild-type and heterozygous littermates. These mice are also fertile. In addition, histological analyses of internal organs did not reveal any apparent differences to heterozygous littermates (data not shown). Thus, in the animal, removal of the SRR of Chd1 is compatible with normal development and life.

### Evidence for niche-dependent compensation mechanisms of Chd1ΔSRR-mediated ESC differentiation defects

**Figure 5:**
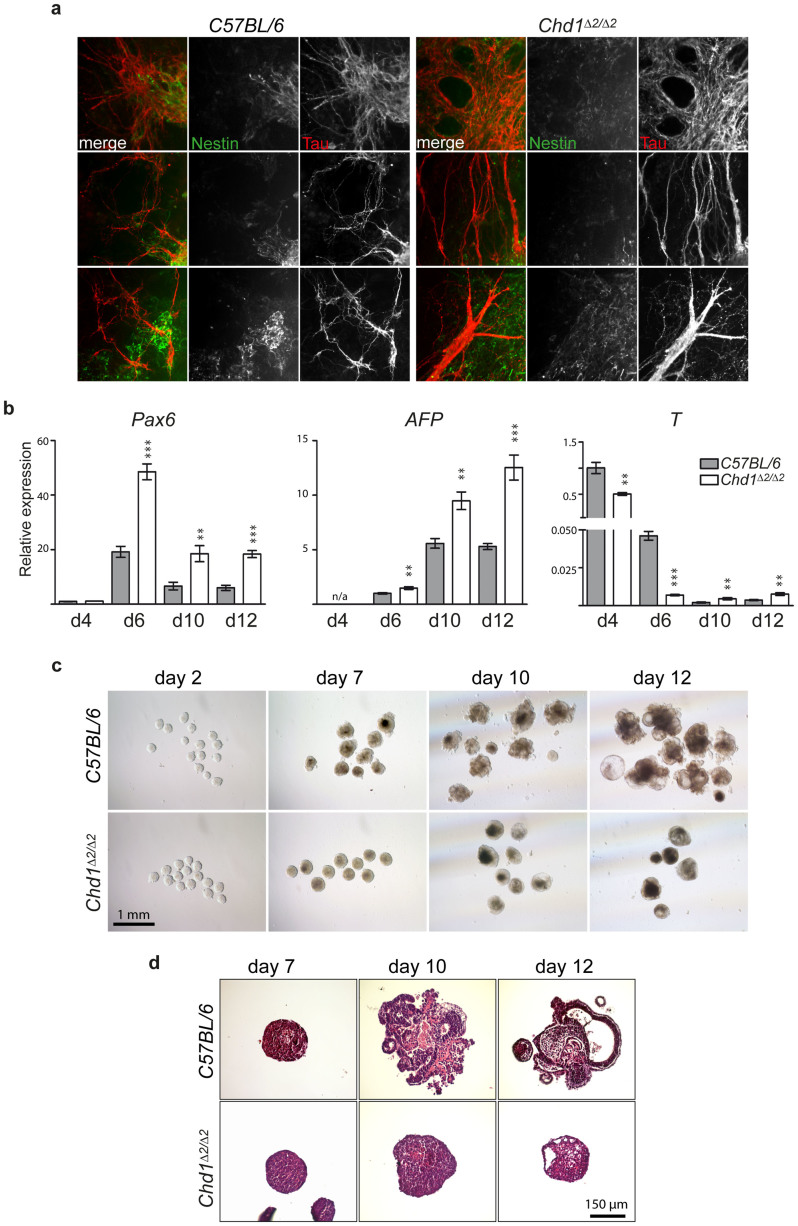
ESCs derived from healthy *Chd1^Δ2/Δ2^* mice show differentiation defects *in vitro*. (a) *Chd1^Δ2/Δ2^* EB outgrowths show enhanced appearance of neural structures. The differentiation of ESCs derived from *Chd1^Δ2/Δ2^* and control C57BL/6NCrl mice and staining for Nestin (green) and Tau (red) was performed as in Figure 4a. (b) Mutant *Chd1* EB colonies exhibit higher levels of the ecto- and endoderm markers *Pax6* and *Afp*, respectively, and they show reduced *T* expression similar to the phenotype of EB colonies derived from *in vitro* generated *Chd1^Δ2/Δ2^* ESCs. RT-qPCR analyses were performed on EB outgrowths as described in Figure 4. (c, d) Decreased morphological complexity of *Chd1^Δ2/Δ2^* EBs compared to control EBs upon *in vitro* differentiation. EBs were maintained in suspension culture and light microscopic images were taken at the indicated time points (c) or EBs were processed for sectioning and H&E staining (d).

To examine if niche-dependent mechanisms might have served to overcome potential developmental defects of *Chd1^Δ2/Δ2^* early embryos, we established several independent ESC lines from *Chd1^Δ2/Δ2^* and C57BL/6NCrl control mice. AP, Oct4 and H3K4me3/H3K9me3 stainings of ESCs revealed no differences between wild-type and mutant ESCs, and stem cell marker gene expression was similarly comparable (Supplementary Figure S7). We then induced EB formation and confirmed a preference for neural differentiation of the newly established *Chd1^Δ2/Δ2^* ESCs that was even more pronounced than in the EBs derived from the *in vitro* generated mutant ESCs (Figure 4a). The cells of the outgrowing mutant EBs exhibited a morphology of neural cells, confirmed by Tau and Nestin stainings, with thick Tau-positive fibers in extended networks (Figure 5a). These more advanced differentiation aspects were not observed in control C57BL/6NCrl EBs (Figure 5a). Gene expression analyses at 4 to 12 days of ESC differentiation revealed that the *Chd1^Δ2/Δ2^* EB outgrowths exhibited increased levels of the neuroectoderm marker *Pax6* and the visceral endoderm marker *Afp*. By contrast, *T* expression was slightly decreased in 4 day old *Chd1^Δ2/Δ2^* EB colonies and considerably lower at day 6 compared to EB colonies derived from control ESCs (Figure 5b). We additionally analyzed the overall morphology of *Chd1^Δ2/Δ2^* and control EBs in suspension culture and found striking differences. While control EBs showed distinct differentiated cell morphologies and extensive cavitation, *Chd1* mutant EBs kept a roundish and more compact shape and exhibited only few and small cysts (Figure 5c and d) even after prolonged differentiation time (18 days, Supplementary Figure S8). Thus, we conclude that the SRR of Chd1 is required for proper differentiation of ESCs *in vitro*, while its loss can be compensated by other as yet unknown mechanisms during embryo development *in vivo*.

## Discussion

Chromatin plasticity is a crucial factor for the maintenance of ESC identity, and factors that affect chromatin dynamics therefore are key players in the regulatory processes that govern self-renewal and differentiation potential. In this work, we have investigated the contribution of the ATP-dependent chromatin assembly and remodeling factor Chd1 to these processes. The study of an N-terminal truncation mutant of Chd1 that lacks a particularly serine-rich domain revealed that although ESC self-renewal ability was not affected by this mutation, differentiation of ESCs was severely compromised by the lack of wild-type Chd1. We observed a marked propensity for the mutant ESCs to differentiate along the neuroectodermal lineage. These observations are similar to the findings by Gaspar-Maia *et al.*^26^, who investigated Chd1 function in ESCs by an RNAi-mediated knock-down strategy. However, there are several notable differences. First, *Chd1* knock-down ESCs displayed lower levels of *Oct4* and increased levels of neural markers such as *Nestin* and *Blbp*. By contrast, our experiments revealed no indication for deregulation of *Oct4* or neural marker genes in ESCs (Figure 3b, c) indicating that the N-terminal portion of the Chd1 protein is not required for maintenance of pluripotency marker levels or repression of differentiation genes.

Second, although knock-down of *Chd1* also resulted in strongly increased formation of neural lineage cells, markers of primitive endoderm and mesoderm were largely absent under those conditions^26^. By contrast, we find that upon differentiation of Chd1ΔSRR-expressing ESCs, neuroectoderm markers (*Otx2, Fgf5, Sox1, Pax6*) and certain endoderm markers (*FoxA2, Sox17, Afp*) were upregulated. We also observed delayed and decreased upregulation of the primitive streak markers *T* and *Eomes*, decreased *Gata4* and *Gata6* expression and decreased levels of *Goosecoid (Gsc)*. Definitive and visceral endoderm cells can be distinguished by the expression of *Sox17* and *Gsc*. While the former is *Sox17^+^Gsc^+^* the latter is *Sox17^+^Gsc^−^*39. Our results suggest that *Chd1^Δ2/Δ2^* EBs may have reduced definitive endoderm differentiation, which might be a consequence of perturbed mesendoderm formation^42^ given the observed decreased expression of the mesendoderm marker *T* and other mesoderm markers (e.g. *Runx2, Flk1, Fabp4*). Decreased *Gata4* and *Gata6* levels in *Chd1^Δ2/Δ2^* EBs point to aberant primitive endoderm formation^43^,^44^ and, indeed, strongly reduced formation of yolk sac-like structures that are generated by primitive endoderm-derived visceral endoderm (Figure 5c, d; Supplementary Figure S8) supports this notion. Apparently contradictory, however, is the observed upregulation of visceral endoderm markers, such as *Afp* and *Sox17*. Interestingly, such a disconnect between morphological phenotype and molecular marker expression has been observed before upon knock-out of integrin β1^45^,^46^. However, we do not know at this point, if integrins are deregulated as a consequence of *Chd1* mutation. Taken together, ESCs expressing Chd1ΔSRR behave similar to *Chd1* knock-down cells with respect to neuroectoderm differentiation but Chd1ΔSRR affects primitive endoderm and mesendoderm formation to a milder extent than abrogation of Chd1^26^.

Finally, *Chd1* RNAi led to increased heterochromatinization in ESCs, and it was suggested that maintaining an open chromatin conformation might serve as a mechanism by which Chd1 regulates pluripotency in ESCs. In our study, Chd1ΔSRR did not cause detectable changes in heterochromatin versus euchromatin distribution (Figure 3d), but defective ESC differentiation still occurred. The lack of changes in chromatin modification patterns in Chd1ΔSRR-expressing cells is perhaps not surprising if viewed in light of recent findings in mammalian cells that suggest a role for Chd1 in suppressing histone turn-over and thus in the conservation of modification marks in gene bodies^47^. Since our *in vitro* results show that deletion of the SRR does not impair chromatin assembly activity (Figure 2e), it is likely that nucleosome reassembly by Chd1ΔSRR in the course of transcription elongation is still possible and thus co-transcriptional H3K4me3 marks can be maintained, which in turn might serve to counteract progressive accumulation of H3K9me3 as observed in *Chd1* RNAi experiments^9^.

Of note, Skene *et al.*^47^ show in their work that Chd1 also functions at promoters to alleviate the nucleosomal barrier impeding RNA polymerase II promoter escape, and they suggest that recruitment of Chd1 to either the promoter or the gene body may involve different mechanisms. We have shown here that Chd1 is a phosphoprotein and that the majority of phosphorylation sites reside in the N-terminal SRR (Figure 2f, g). Deletion of exon 2 results in the removal of roughly two thirds of potential phosphorylation sites from the N-terminus. Thus, while this protein part does not affect the chromatin assembly activity of Chd1, it is possible that it is required for the interaction with factors involved in the recruitment of Chd1 to the transcriptional start site (TSS) while being less important for the recruitment to the gene body. This might lead to a deregulation of target genes (such as developmental genes) without affecting overall histone methylation levels.

While mammalian Chd1 localizes to hundreds of active genes^26^,^47^, the individual mode of recruitment may render some genes more sensitive to defects in the Chd1 protein than others. For example, our results show upregulation of *Noggin* in *Chd1^Δ2/Δ2^* ESCs as well as in the first stages of differentiation (Figure 4e, Supplementary Figure S5). This may direct cells towards preferential differentiation into ectodermal cell types and suppress or delay mesodermal fate^48^. ESC populations are known to exhibit a degree of heterogeneity with respect to the expression of regulatory and signaling factors^49^, and a slight skewing of the expression levels of certain factors (such as *Noggin*) as a consequence of not fully functional Chd1 might be amplified by feedback mechanisms^50^ culminating in altered cell fate decisions. The fact that deletion of exon 2 of *Chd1* has no obvious developmental consequences *in vivo*, yet newly established ESCs derived from the *Chd1^Δ2/Δ2^* mice also exhibited *in vitro* differentiation defects, gives support to the idea for a deregulation of signaling pathway components in the Chd1ΔSRR mutant ESCs.

The discrepancy between the *in vivo* and *in vitro* behavior of *Chd1^Δ2/Δ2^* cells may be explained by the greatly differing microenvironments ESCs and inner cell mass cells of the developing embryo are exposed to. It is conceivable that differences in the spatial and temporal expression of growth factors in the blastocyst may counteract any deregulation of developmental genes that is caused by SRR deletion of Chd1. For example, moderately altered levels of *Noggin*, as observed in *Chd1^Δ2/Δ2^* ESCs, may be buffered *in vivo* e.g. by increased BMP levels or by compensatory induction of other remodeling factors, such as the closely related Chd2 that shares biochemical features with Chd1 and was shown to cause developmental delay in a gene-trap mouse model^51^,^52^, whereas such compensatory pathways may be restricted in the artificial cell culture environment.

Alternatively, the effects of aberrant chromatin or transcriptional regulation caused by partially functional Chd1ΔSRR protein could be subtle and could require several cell generations to reach a threshold level at which defects become obvious. It was recently shown that chromatin remodeling factors can localize to many overlapping sites in the genome and that chromatin accessibility at a particular site may be governed by agonistic and antagonistic effects of different remodelers^53^. It is, for example, possible that the presence of mutant Chd1 disturbs the equilibrium of chromatin remodeling at particular genes leading to accumulation of an unwanted chromatin state and consequently transcriptional deregulation over time. The fact that *in vitro* cultivated ESCs are usually kept in culture for many passages in contrast to ICM cells that for the most part transit into lineage committed cells comparably quickly may be responsible for progressively increasing localized chromatin changes which manifest in the altered differentiation behavior of *Chd1^Δ2/Δ2^* ESCs *in vitro* versus *in vivo*. While we have kept the passage number of our *in vitro* generated mutant ESCs as low as possible, we cannot rule out that such an accumulation of defects has occurred. However, we did not detect an increase in H3K9 methylation or transcriptional deregulation of ESC marker genes in mutant or control ESCs that were passaged at least 12 times (data not shown).

Altogether, our results support a role for Chd1 as an early regulator of ESC differentiation and they show that the phosphorylated serine-rich N-terminal region is required for full activity of Chd1 in this process.

## Methods

### Generation of mutant ESCs and *Chd1^Δ2/Δ2^*mice

*Chd1^flox/flox^* ESCs were generated by mutation of the *Chd1* gene in ESCs using homologous recombination. Plasmids pDONR-P4-P1r, pDONR-P2r-P3 (Invitrogen), pENTR-frt-Neo-frt-loxP and pDEST-DTA-MLS^54^ were used to generate the targeting vector pK1-Chd1 using the Multisite Gateway® Technology (Invitrogen). The *Chd1* homologous sequences range from 1873 bp upstream of exon 1 to 5552 bp downstream of exon 2. A loxP site was inserted in intron 1 1077 bp upstream of exon 2, while a G418 selection cassette containing an additional loxP site at its 3′ end was inserted 799 bp downstream of exon 2. In addition, a negative selection marker (*DTA*) gene was included to select against ectopic integration events. The targeting vector was transfected into mouse ESCs (129S2/SvPasCrl) and homologous recombination events were detected by Southern blot analysis. Positive ESC clones were adapted to growth on gelatin-coated plates and subjected to two rounds of high concentration G418 selection (5 mg/ml for 7 days) to select for *Chd1^flox/flox^* ESCs generated by mitotic recombination. *Chd1^flox/flox^* ESCs were transfected with the pANMerCreMer plasmid, and Cre-positive clones were treated with 4 µM tamoxifen for 5 days. The homozygous deletion of *Chd1* exon 2 (*Chd1^Δ2/Δ2^*) was verified by PCR and Southern blot analyses.

To generate *Chd1^Δ2/Δ2^* mice the pK1a-Chd1 targeting vector (identical to pK1-Chd1 except that the 3′ LoxP site was inserted 1433 bp upstream of exon 2) was transfected into Bruce4 ESCs and clones containing homologous recombination events were injected into CD-1 blastocysts. Chimeras were crossed with albinoB6 mice to obtain F1 generation carrying the *Chd1^flox^* allele. The *Chd1^flox/+^* mice were further backcrossed to C57BL/6NCrl mice. Exon 2 deletion was achieved by crossing *Chd1^flox/+^* mice with *Ubi-Cre* mice followed by crosses between the resulting *Chd1^Δ2/+^* mice. Genotyping of animals was performed by PCR with primers P1 (5′-CAGCCTTAGGATCTGTGGTAAAGC) and P3 (5′-CGCATCGCCTTCTATCGCCTTC) for detecting the floxed allele and with primers P4 (5′-GCGAGGCTGGAAAGGAAGTC) and P2 (5′-CCTCCAGCTTAGAGACTCAC) for detecting the deletion of *Chd1* exon 2.

### *In vitro* differentiation of ESCs

ESC were cultured in ESC-2i/LIF medium (DMEM high glucose with GlutaMAX^TM^-1 (Gibco), 20% ES cell tested FBS (Gibco), 1 x NEAA (Gibco), 0.05 mM β-mercaptoethanol, 12.5 µg/l LIF, 3 µM CHIR99021 (Axon Medchem) and 1 µM PD0325901 (Axon Medchem)). Differentiation of ESCs was performed by induction of embryoid body (EB) formation according to the hanging drop method^55^. 5 × 10^4^ cells were suspended in 1 ml differentiation medium (ESC medium without 2i/LIF) and drops of 20 µl cell suspension were transferred to the lid of a bacterial grade petri dish. Hanging drops were incubated for 2 days and subsequently 20-30 EBs were plated per well in 6-well culture dishes coated with 0.2% gelatin. Outgrowing EBs were further cultured for 12 days in differentiation medium with 10% FBS and medium changes every 2 days. Alternatively (Figure 5c), EBs were generated by transferring hanging drops to ultra low attachment plates (Corning) 2 days after initial seeding and further culturing in differentiation medium to the time points indicated in the Figure 5c. EBs derived in this way were collected, fixed with 4% paraformaldehyde for 2 hrs at 4°C, dehydrated and embedded in paraffin for sectioning. 7 µm sections were stained with hematoxylin and counterstained with eosin.

### ESC isolation from mouse blastocysts

Female mice (C57BL/6NCrl and *Chd1^Δ2/Δ2^*) were sacrificed at 3.5 dpc and dissected under sterile conditions. Blastocysts were collected by flushing the uteri with PBS/10% FBS and subsequently transferred into drops of Tyrode's solution (Sigma) until the *zona pellucida* was no longer visible. Blastocysts were then transferred individually onto feeder cells in ESC-2i/LIF medium. Once the ICM outgrowth was evident, the cells were dissociated and re-plated onto feeder cells in ESC medium to expand the ESC population. The quality of each ESC clone was determined by morphology evaluation and by measuring the expression of several ESC marker genes by RT-qPCR.

### *In vitro* transcription/translation

Templates for *in vitro* transcription were generated by PCR with *Chd1*-specific forward primers containing a T7 promoter overhang and reverse primers containing a Flag-tag encoding sequence. The different templates are schematically depicted in Figure 2a. Primer sequences are available upon request. *In vitro* transcription/translation reactions were performed using the TNT® Quick Coupled Transcription/Translation System (Promega) according to the manufacturer's instructions. Briefly, 600 ng of PCR-generated template was used in a 25 µl reaction in the presence of 10 µCi ^35^S-methionine. After incubation for 2.5 h at 37°C, 10 µl of reaction were denatured at 70°C for 10 min and separated by SDS-PAGE. After fixation and drying the gel was exposed to a Fuji imaging screen and signal was detected using a STORM 840 scanner (Molecular Dynamics).

### Synthesis and purification of recombinant proteins

Recombinant Chd1 proteins were produced using the baculovirus system (Bac-to-Bac®, Life Technologies). The coding sequences of full-length mouse Chd1 (aa 1-1711), and two truncated versions of Chd1 (aa 101-1711 and aa 150-1711) were cloned using the in-Fusion PCR Cloning Kit (Clontech) into the *Sal*I site of a Flag-tag encoding pFastBac1 vector^24^. Bacmid preparation, generation of virus stocks and infection of *Sf9* cells were performed according to the manufacturer's instructions. For the purification of Flag-tagged Chd1 proteins, ~2 × 10^8^ *Sf9* cells were infected with the amplified virus stock and allowed to express protein for 3 days. Cells were collected, washed with PBS and resuspended in 10 ml Lysis Buffer (20 mM Tris/HCl pH 7.9, 10 mM NaCl, 4 mM MgCl_2_, 0.4 mM EDTA, 20% glycerol, 0.4 mM PMSF, 2 mM DTT). Cell disruption was performed by occasional douncing in a glass douncer over 30 min on ice. NaCl was then added to a final concentration of 500 mM and cell debris was removed by centrifugation. The supernatant was diluted 1:1 with Dilution Buffer (20 mM Tris/HCl pH 7.9, 10% glycerol, 0.02% NP-40) and incubated with 200 µl slurry of Flag-M2 agarose (Sigma) for 4 h on a rotating wheel at 4°C. Flag-beads were collected by centrifugation, extensively washed with Wash Buffer (20 mM Tris/HCl pH 7.9, 150 mM NaCl, 2 mM MgCl_2_, 0.2 mM EDTA, 15% glycerol, 0.2 mM PMSF, 1 mM DTT) and repeatedly eluted with 150 µl Elution Buffer (Wash Buffer + 0.4 mg/ml Flag-peptide). Recombinant Nap1 was generated as described previously^24^.

### Chromatin assembly assays

*In vitro* chromatin assembly assays and analysis of reaction products by DNA supercoiling assay were performed as described previously^24^ with the following modifications: Typically, 20 nM of wild-type or mutant Chd1 protein was used and reactions were incubated for 70 min at 35°C.

### Protein extract preparation and western blotting

Whole cell protein extracts from ESCs were prepared by homogenizing cells in standard RIPA buffer supplemented with PIC protease inhibitors (Roche), 20 U/ml DNase I and 0.2 mg/ml RNase A. Lysates were clarified by centrifugation at 14,000 rpm. Proteins were separated by SDS-PAGE, transferred to nitrocellulose membrane and incubated with antibodies against mouse Chd1 (Proteintech 20576-1-AP; 1:1000), alpha-tubulin (Sigma, T-5168; 1:8000), H3K4me3 (Diagenode 003-050, 1:1000) or H3K9me3 (Diagenode 0650-050, 1:1000).

### Immunofluorescence microscopy

For immunofluorescence staining ESCs were grown on coverslips coated with gelatin or Geltrex (Life Technologies) before fixation for 10 min in 5% paraformaldehyde/0.3% Triton X-100/PBS. Fixed cells were washed twice with PBS, blocked in 1% BSA/PBS for 1 h and incubated overnight at 4°C with primary antibodies in 1% BSA/PBS. The following primary antibodies were used: mChd1 (Proteintech 20576-1-AP, 1:300) H3K4me3 (Diagenode 003-050, 1:250), H3K9me3 (Diagenode 0650-050, 1:250), Nestin (Santa Cruz SC33677, 1:200), Tau (Dako A0024, 1:1000), Oct4 (Santa Cruz Sc5279, 1:200). Secondary antibodies coupled with Alexa 488 or 594 (Molecular Probes) were incubated for 1 h at room temperature. Alkaline phosphatase stainings were performed with the AP Detection Kit (Millipore) according to the manufacturer's instructions. Microscopic images were taken either on a ZEISS Axioplan microscope or on a Leica TCS SP5 confocal microscope and processed using AxioVision Rel. 4.8 or LAS AF Lite software.

### RT-qPCR analyses

Total RNA was isolated from ESCs or EBs at different time points and cDNA was synthesized as described previously^28^. Real-time PCR was performed using POWER SYBR Green PCR mastermix (Applied Biosystems) with 1.25 ng/µl cDNA and 0.8 µM primers in a StepONE Plus Instrument (Applied Biosystems). Primer sequences are available upon request. Data were normalized against *Gapdh* and differences between wild-type and mutant samples were calculated using the ΔΔC_T_ method. Statistical significance of differences of at least 3 biological replicates was determined using unpaired t-test (Prism 5.0).

## Author Contributions

P.P. and A.L. designed the study and analyzed the data; P.P., C.Y.L., R.N., C.S., Y.T.S. and M.M. performed experiments; A.V., S.G., R.N. provided reagents and contributed intellectual input; P.P., C.Y.L. and A.L. wrote the manuscript. All co-authors critically reviewed the manuscript.

## Supplementary Material

Supplementary Information Supplementary Information
