# Supplementary material for: Embryonic stem cell differentiation requires full length Chd1
Source: Sci Rep. 2015 Jan 26;5:8007. doi: 10.1038/srep08007 (PMC4306112; doi:10.1038/srep08007)
Supplement: Supplementary Information [file srep08007-s1.pdf]

**Supplementary Information**

## Embryonic stem cell differentiation requires full length Chd1

Paolo Piatti[1], Chin Yan Lim[2], Roxana Nat[3], Andreas Villunger[4], Stephan Geley[5], Yan Ting Shue[2], Claudia Soratroi[4], Markus Moser[6], Alexandra Lusser[1*]

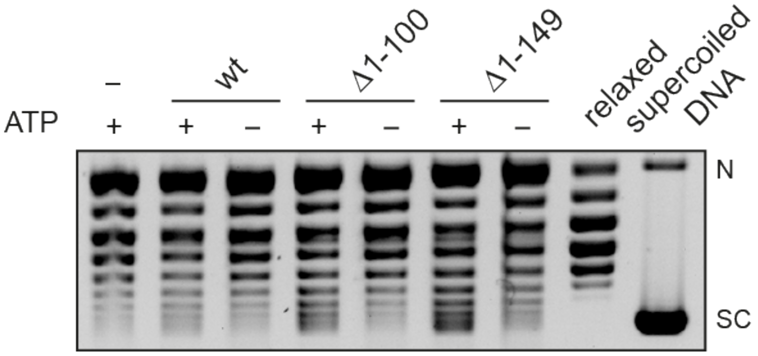


**Supplementary Figure S1:** N-terminally truncated Chd1 is capable of efficient ATP-dependent nucleosome assembly. Products of nucleosome assembly reactions with wt, Δ1-100 and Δ1-149 proteins (20 nM) were analyzed for DNA supercoiling efficiency in the presence and absence of ATP. Reference lanes contain topoisomerase I-relaxed (*relaxed*) and supercoiled plasmid DNA (*supercoiled*), respectively. The positions of nicked (N) and fully supercoiled (SC) DNA species are indicated.

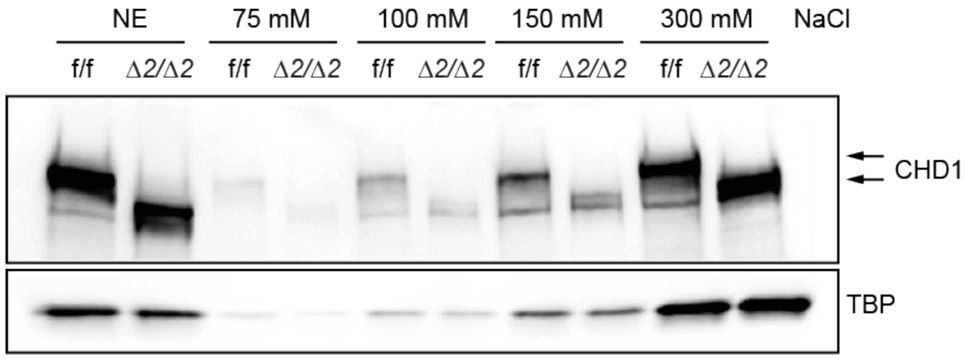


**Supplementary Figure S2: Extraction of wild-type Chd1 and Chd1ΔSRR from ESC nuclei requires similar salt concentrations.** Nuclei were prepared from $3x10^7$ cells each of the *Chd1*$^{Δ2/Δ2}$ (Δ2/Δ2) and *Chd1*$^{flox/flox}$ (f/f) ESC lines, divided into 5 identical batches and extracted with buffers containing increasing NaCl concentrations (indicated above the lanes). As a reference, one batch was extracted using RIPA buffer containing DNAseI and RNaseA (lanes "NE"). All extracts were separated by SDS-PAGE and transferred onto nitrocellulose membranes for immunodetection with antibodies against Chd1 and TATA-binding protein (TBP) as a loading control. Arrows indicate the bands for full length Chd1 from wild-type ESCs and truncated Chd1 from *Chd1*$^{Δ2/Δ2}$ ESCs.

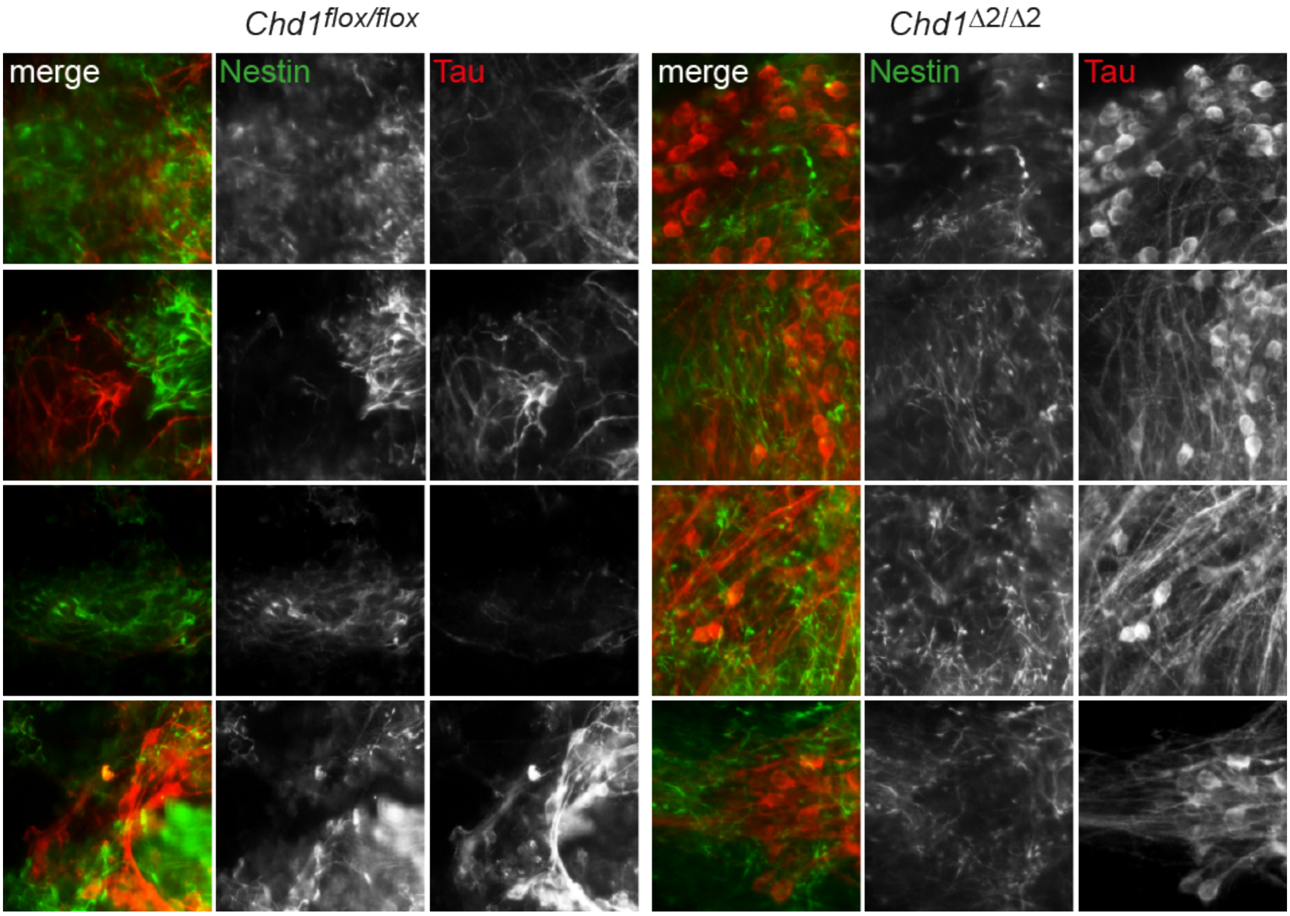


**Supplementary Figure S3: Outgrowing embryoid bodies (EBs) from *Chd1*$^{\Delta 2/\Delta 2}$ cells show increased emergence of neuronal structures compared to *Chd1*$^{flox/flox}$ EBs.** EBs were plated on gelatin-coated dishes and stainings with the neuronal progenitor marker Nestin (green) and the neuronal marker Tau (red) were performed at day 14 after plating. Several example images are shown.

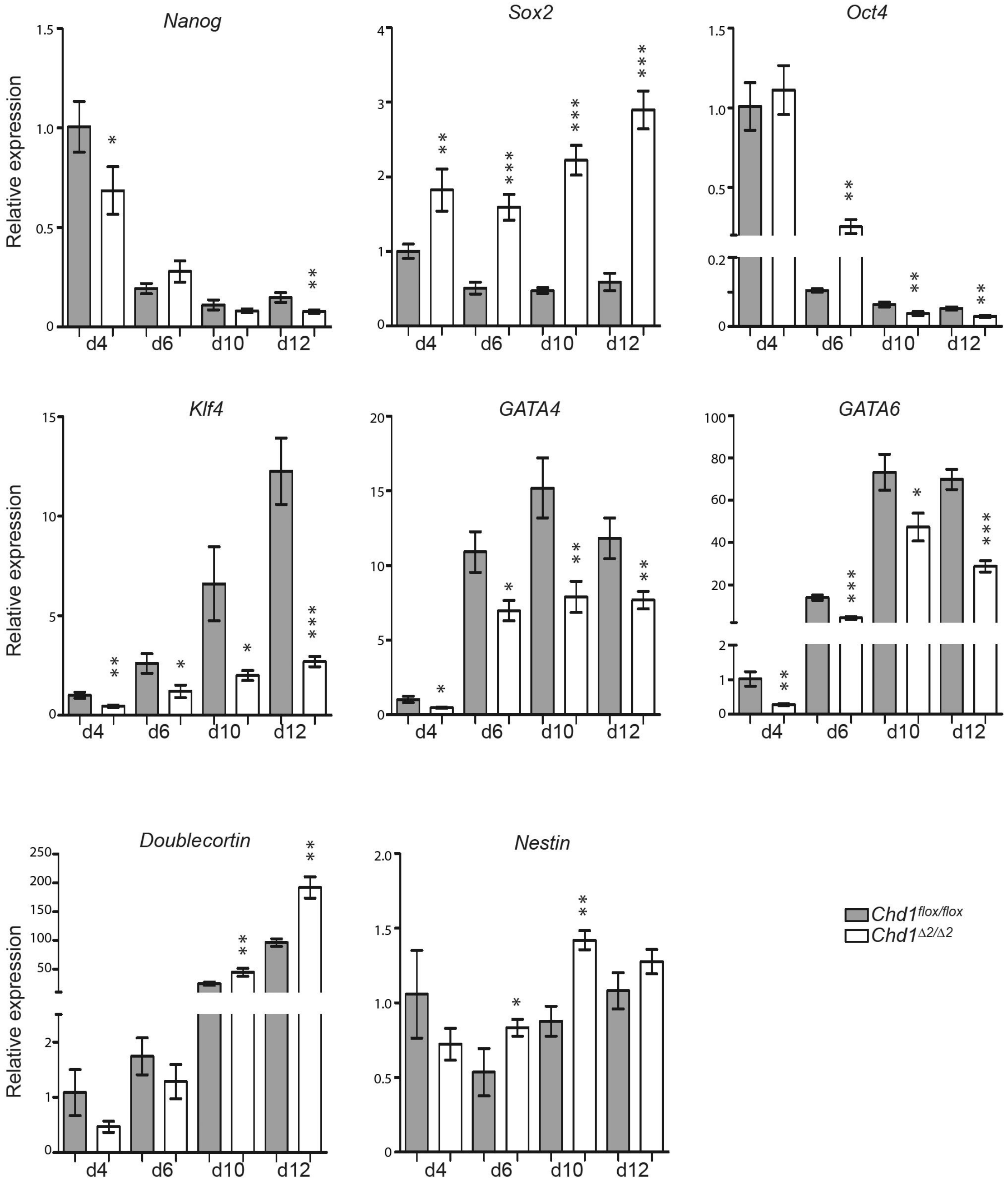


**Supplementary Figure S4. *Chd1$^{Δ2/Δ2}$* EBs show aberrant expression of pluripotency and differentiation genes.** Real time PCR was performed on cDNA derived from *Chd1$^{flox/flox}$* and *Chd1$^{Δ2/Δ2}$* EBs at the indicated time points of outgrowth on gelatin-coated plates and the expression levels of the pluripotency markers *Nanog*, *Oct4*, *Sox2* and *Klf4*, the early endoderm markers *Gata4* and *Gata6* as well as the neureoctoderm markers *Doublecortin* and *Nestin* were determined. Transcript levels were normalized against *Gapdh* and are expressed relative to those of control EB outgrowths at day 4 (*Chd1$^{flox/flox}$*). Values represent mean +/- SD of 2-3 experiments each with EB outgrowths derived from two independent *Chd1$^{flox/flox}$* and *Chd1$^{Δ2/Δ2}$* ESC clones (*p<0.05; **p<0.001; ***p<0.0001).

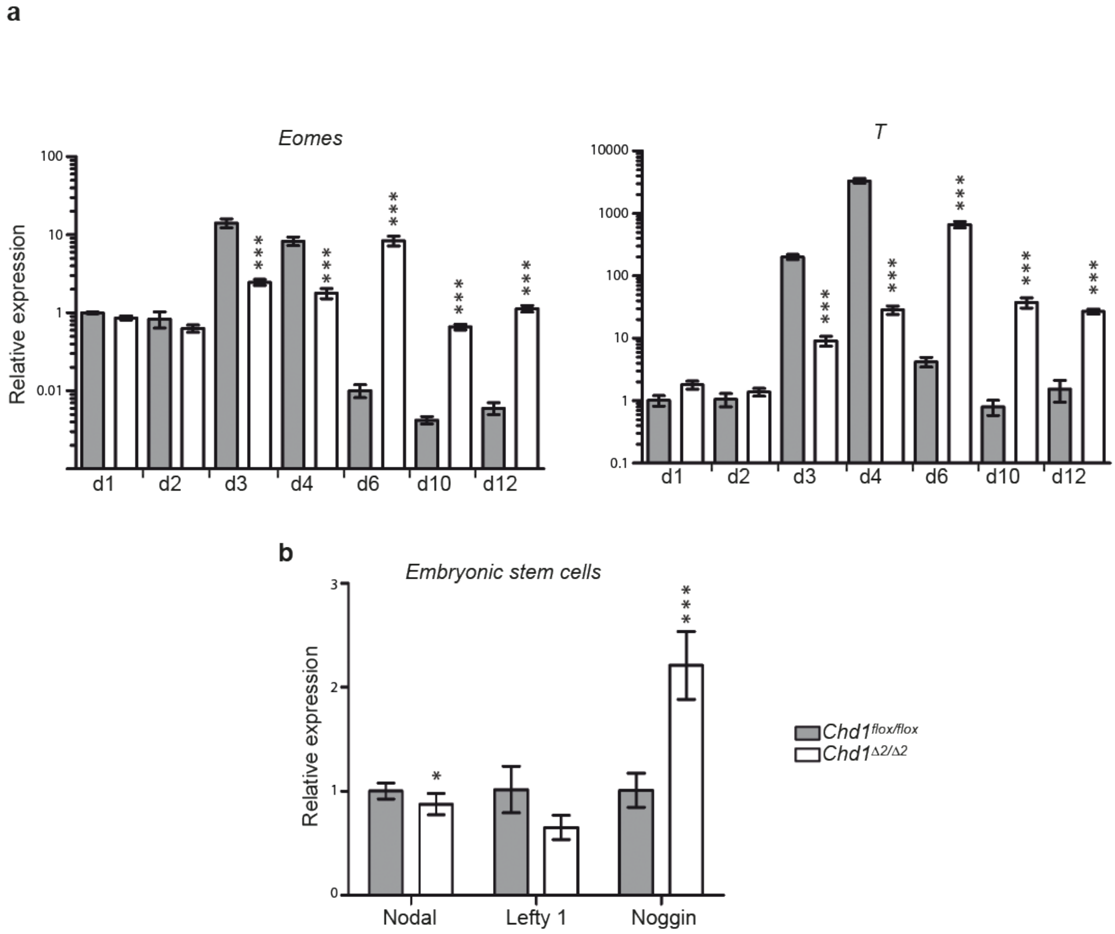


**Supplementary Figure S5: Delayed expression of *T* and *Eomes* in outgrowing *Chd1*$^{\Delta 2/\Delta 2}$ EBs and increased Noggin levels in *Chd1*$^{\Delta 2/\Delta 2}$ ESCs. (a)** A time course expression analysis of *Eomes* and *T* was performed on *Chd1*$^{flox/flox}$ and *Chd1*$^{\Delta 2/\Delta 2}$ EB outgrowths at the indicated time points. Both genes show similarly delayed expression dynamics in *Chd1*$^{\Delta 2/\Delta 2}$ compared to *Chd1*$^{flox/flox}$ cells. **(b)** The expression of the BMP signaling pathway inhibitor *Noggin* is significantly upregulated in *Chd1*$^{\Delta 2/\Delta 2}$ ESCs. **(a, b)** Transcript levels were normalized against *Gapdh* and are expressed relative to those of control EBs (*Chd1*$^{flox/flox}$) at day 1 (**a**) or control ESCs (**b**). Values represent mean +/- SD of 2-3 experiments each with EB colonies (**a**) or ESCs (**b**) derived from two independent *Chd1*$^{flox/flox}$ and *Chd1*$^{\Delta 2/\Delta 2}$ ESC clones (*p<0.05; **p<0.001; ***p<0.0001).

**a**

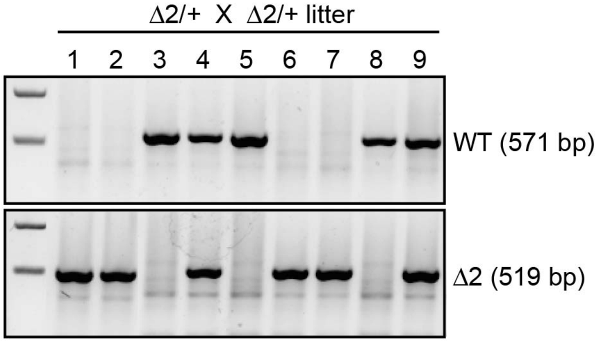


**b**

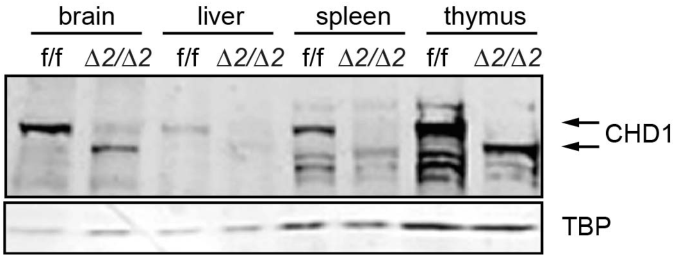


**Supplementary Figure S6**: **Truncated Chd1 is expressed in all analyzed tissues of *Chd1*$^{\Delta2/\Delta2}$ mice.** (a) PCR genotyping data from a representative litter of a heterozygous *Chd1*$^{\Delta2/+}$ intercross. The wild-type allele (WT) was amplified using primers P1 and P2 (see materials and methods), the exon 2 deletion allele (Δ2) was detected with primers P4 and P2. (b) Immunoblot analysis with antibodies against Chd1 reveals expression of N-terminally truncated Chd1 in brain, spleen, liver and thymus of *Chd1*$^{\Delta2/\Delta2}$ mice (Δ2/Δ2). For comparison protein extracts of the same organs from a homozygous floxed mouse were analyzed (f/f). Truncated and full-length Chd1 bands are indicated by arrows. TBP was used as a loading control.

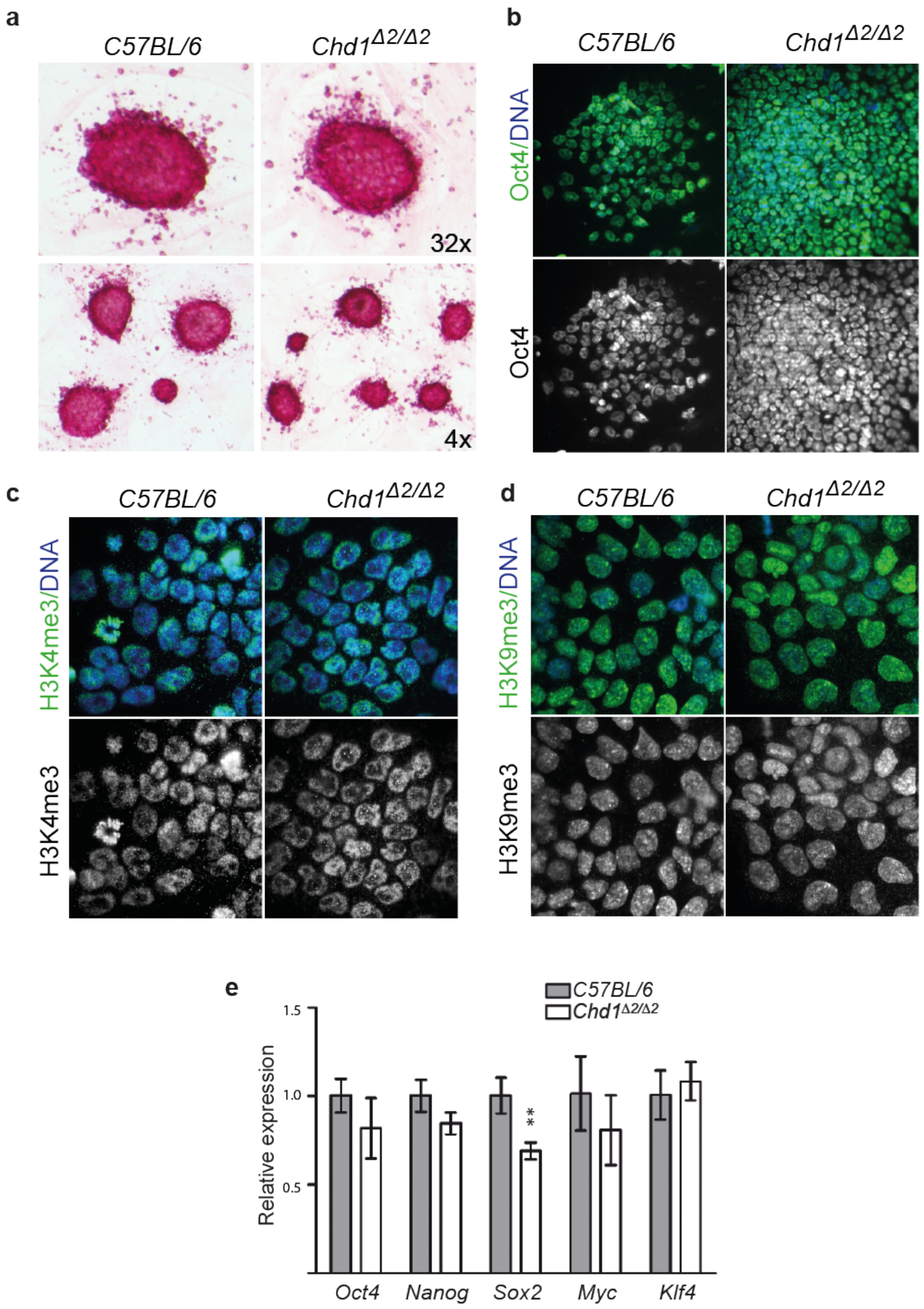


**Supplementary Figure S7**: **Cellular and molecular characterization of newly established ESC clones.** (a) Alkaline phosphatase (AP) staining of mutant and control ESC clones shows uniform staining in all colonies. Upper panels, single ESC colony; lower panels; lower magnification images showing multiple colonies. (b) Staining of ESCs with Oct4 (green) reveals expression of this pluripotency marker in all cells. DNA was visualized by DAPI staining. (c, d) Comparable distribution of H3K4me3 (c) and H3K9me3 (d) in newly established ESCs. DNA was visualized by DAPI staining. (e) RT-qPCR analysis of different pluripotency markers reveals similar expression of *Oct4*, *Nanog*,

*Myc* and *Klf4* and slightly reduced *Sox2* levels in mutant ESC clones derived from *Chd1*$^{\Delta 2/\Delta 2}$ mice compared to ESCs derived from C57BL/6NCrl mice. Transcript levels were normalized against *Gapdh* and are expressed relative to those of control ESCs. Values represent mean +/- SD of two independent ESC clones each (**p<0.001).

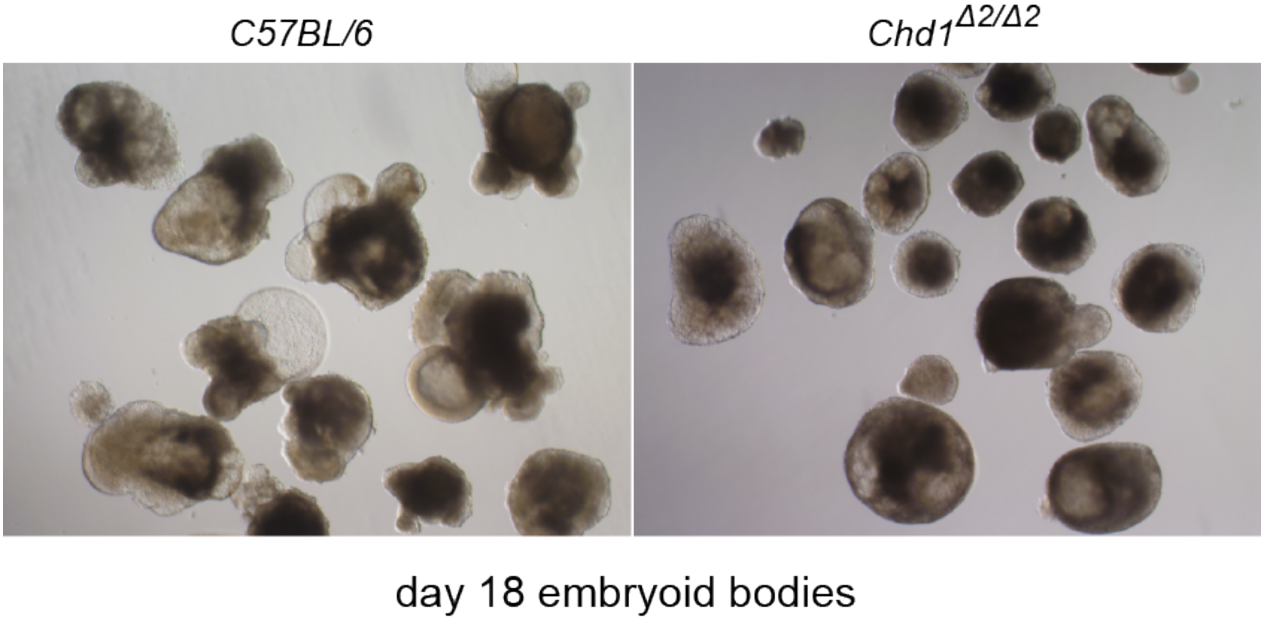


**Supplementary Figure S8**: **Embryoid body induction results in less complex morphology of EBs derived from *Chd1*$^{Δ2/Δ2}$ mice compared to those from control mice.** EB induction was performed using the hanging drop method and EBs were grown for 18 days.
